# Development of a flow-free magnetic actuation platform for an automated microfluidic ELISA†

**DOI:** 10.1039/c8ra07607c

**Published:** 2019-03-12

**Authors:** Chad Coarsey, Benjamin Coleman, Md Alamgir Kabir, Mazhar Sher, Waseem Asghar

**Affiliations:** Asghar-Lab, Micro and Nanotechnology for Medicine, College of Engineering and Computer Science Boca Raton FL 33431 USA wasghar@fau.edu; Department of Computer & Electrical Engineering and Computer Science, Florida Atlantic University Boca Raton FL 33431 USA; Department of Biological Sciences, Florida Atlantic University Boca Raton FL 33431 USA

## Abstract

There is a need to create an easily deployable and point-of-care (POC) diagnostic platform for disease outbreaks and for monitoring and maintenance of chronic illnesses. Such platforms are useful in regions where access to clinical laboratories may be limited or constrained using cost-effective solutions to quickly process high numbers of samples. Using oil and water liquid–liquid interphase separation, immunoassays developed for microfluidic chips can potentially meet this need when leveraged with electromagnetic actuation and antibody-coated superparamagnetic beads. We have developed a microfluidic immunoassay detection platform, which enables assay automation and maintains successful liquid containment for future use in the field. The assay was studied through a series of magnetic and fluid simulations to demonstrate these optimizations, and an optimized chip was tested using a target model for HIV-1, the p24 capsid antigen. The use of minimal reagents further lowers the cost of each assay and lowers the required sample volume for testing (<50 μL), that can offer easy turnaround for sample collection and assay results. The developed microfluidic immunoassay platform can be easily scaled for multiplex or multi-panel specific testing at the POC.

## Abstract 2

A flow-free device is developed for automated and rapid ELISA testing at the point-of-care settings.

## Graphical abstract

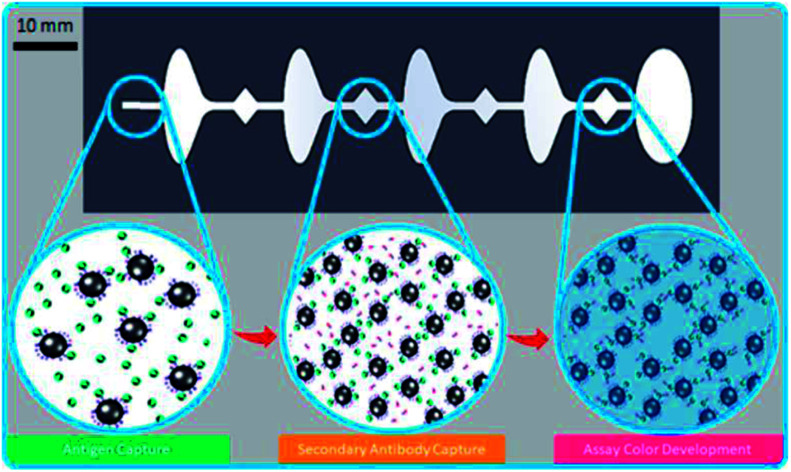

## Introduction

Several emerging and existing pathogens in resource-limited regions pose diagnostic challenges, logistically complicating the ability to identify common outbreaks or endemics including viruses like Ebola^1,2^ and from mosquito-borne illnesses such as Zika virus,^3,4^ malaria^5,6^ and other infectious pathogens.^7^ The access to available healthcare and desire to enable decentralized routine testing in resource-limited regions is a vast challenge that prompts the further need for development of point-of-care (POC) diagnostics, which can be readily deployed, automated, require minimal skills or techniques that any person could utilize and can leverage faster assays than current standards.^8–10^ The ability to rapidly diagnose and render treatment is paramount to disease prevention^11^ and patient health, which demonstrates the value in developing POC testing to identify new patients for treatment.^12–14^ This decentralized POC testing scheme potentially eliminates the need to bring patients to the centralized high-tech labs and enables routine monitoring of chronically diseased or high-risk patients at POC settings such as in local labs or clinics in distant villages. One standard serological detection method is the antigen-capture Enzyme-Linked Immunosorbent Assay (ELISA); which can be found in several variations specific to the target of interest. Several challenges arise for these techniques at the POC as the assay can take up to 12 hours and requires expensive spectrophotometers and trained laboratory personnel. Paper based later flow assays (LFAs) are also developed for disease diagnostics,^10^ however LFAs show poor sensitivities that limit their widespread applications.^10,15,16^ Therefore, new POC diagnostics for deployable and decentralized testing are needed to help quickly diagnose and break the disease chain cycle.^17^ Such challenges are met by miniaturization of the current diagnostic standards, adapting newer tools such as microfluidics to establish assays,^18–23^ and leveraging widespread technology such as smartphones^24–27^ to potentially bridge gaps in global health, and to provide powerful epidemiological tools.^28–32^ Previously, magnetic bead-based microfluidic-ELISA systems were implemented for POC detection of *M. tuberculosis*^33^ and for counting human CD4^+^ cells^34^ which are commonly monitored in routine HIV/AIDS testing to help assess patient immune health. These assays take significantly less time than traditional ELISA as magnetic beads have increased surface area as compared to the flat surface of a well plate, and can be controlled easily using external magnets as previously demonstrated to capture biotargets.^34^

In this paper, using HIV-1 p24 antigen as a model, an automated magnetic bead-based microfluidic ELISA platform was developed for the POC. We tested the developed platform at the clinically accepted values for HIV-1 p24 at a low (20 pg mL^−1^) and middle range targets (60 pg mL^−1^) found in 4^th^ generation combination ELISA kits.^35^ The developed platform provides a flow-free design, thus does not require syringe pumps or other peripherals to maintain the flow. Instead, it uses a low-cost printed platform with external magnet actuation and is controlled using an Arduino Uno microcontroller.

## Methods

### Microfluidic chip design

**Fig. 1:**
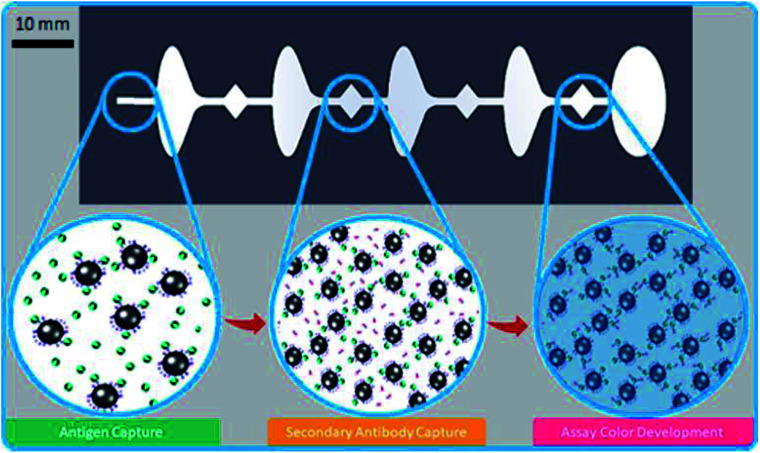
The microfluidic ELISA schematic for automated point-of-care detection of HIV-1; the antigen is first captured in primary well using antibody-coated superparamagnetic beads. The beads move through a washing phase, and then are mixed with a secondary antibody. After a final rinsing stage, the beads are moved and reacted with the color substrate in the final reaction well. The reaction is halted once the beads are pulled into the final oil retention well.

**Fig. 2:**
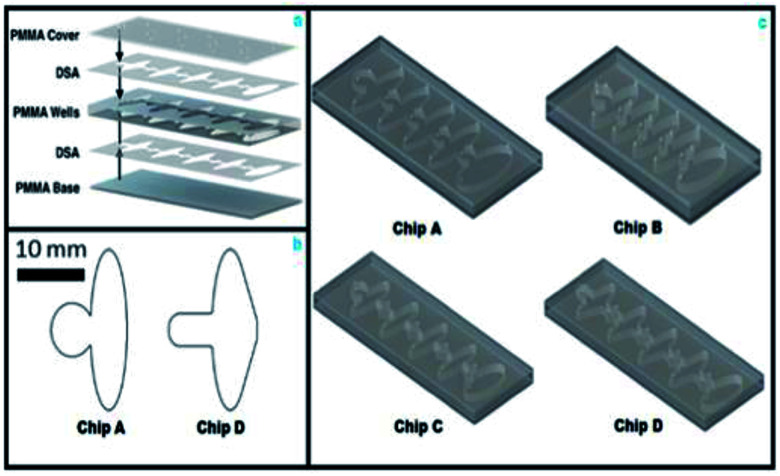
(a–c) The comparison of optimized microfluidic chip designs for automation; (a) the microfluidic chip layout schematic; the microfluidic chip was assembled using three laser cut optically-clear acrylic plastic (PMMA) layers. The PMMA sheets were held together to form the 3-D microfluidic chamber using double-sided adhesive (DSA); (b) the comparison of initial well chip geometry between Chip A and Chip D to optimize bead paramagnetization; (c) the chip design schematic of progressive chip optimizations; the comparison of optimized microfluidic chip dimensions; chip prototypes A–D (pictured above) and displays their respective layer thickness and volumetric dimensions. Chip D represents the prototype used in the microfluidic ELISA test.

The microfluidic chip was designed with diamond aqueous reagent wells separated by elliptical mineral oil wells (Fig. 1). At the end of the chip, a large oil phase allowed the beads to exit the final aqueous well, so they did not interfere with colorimetric evaluation. The chip design was made of three layers consisting of a top layer with inlets, a middle layer with micro-wells, and a solid bottom layer (Fig. 2a). The pipette inlets were cut using a laser cutter to be at 0.4 mm diameter, which allowed easy access to each of the wells for reagent loading. There was also a counterhole made at the same diameter to allow for air to escape, as the wells fill with their respective reagent. The holes were small enough to retain the liquids for more than 60 minutes and were able to contain the aqueous reagents through hydrophobic interaction with the plastic and curvature of the optimized inlet. The wells had distinct shapes to optimize the interaction with each reagent. The oval shapes were reserved to the oil separation wells, the curvature of the wells allows for continuous smooth flow near the aqueous well junctions and lessens fluid turbulence. The aqueous reagent wells had diamond shapes to allow for quick hydrophobic interactions (from the water repelling the PMMA well wall) that facilitates the filling. The chips were held together with double-sided adhesive (DSA), which were cut to match the well pattern on the middle layer, aligned with the well pattern and then pressed in a vice. A soft mallet was used to eliminate any remaining imperfections or air bubbles in the DSA adhesion. The chip and well dimensions were designed to eliminate bead retention during transfer from one well to the other and to improve the interface stability. The geometry of the chip and phase interfaces was optimized experimentally with an iterative design procedure. Fig. 2b shows the changes in bead capture wells to optimize the initial paramagnetization of the bead pellet. These dimensions were modified to accommodate magnetic and microfluidic design constraints, demonstrated in the prototype chips A–D (Fig. 2c) (see ESI Table 1† for chip thickness and volumetric dimensions).

The microfluidic chips were manufactured from polymethylmethacrylate (PMMA) 1′ × 1′ sheets using the VLS 2.3 CO_2_ laser cutter (Universal Laser). To provide additional insight on the microfluidic and magnetic design problem, we simulated each design change using COMSOL Multiphysics software. We modeled the magnetic field strength for the neodymium magnets and found the magnetic force (ESI eqn (1)†) on the beads for each design and solved the Navier–Stokes equations (ESI eqn (2)†) for laminar flow of immiscible phase interfaces for a variety of chip geometries. The PMMA is inexpensive and many chips can be made through use of laser cutting for rapid prototyping and fabrication of chips in a matter of minutes (see ESI Table 2†).

### Platform automation and bead control

**Fig. 3:**
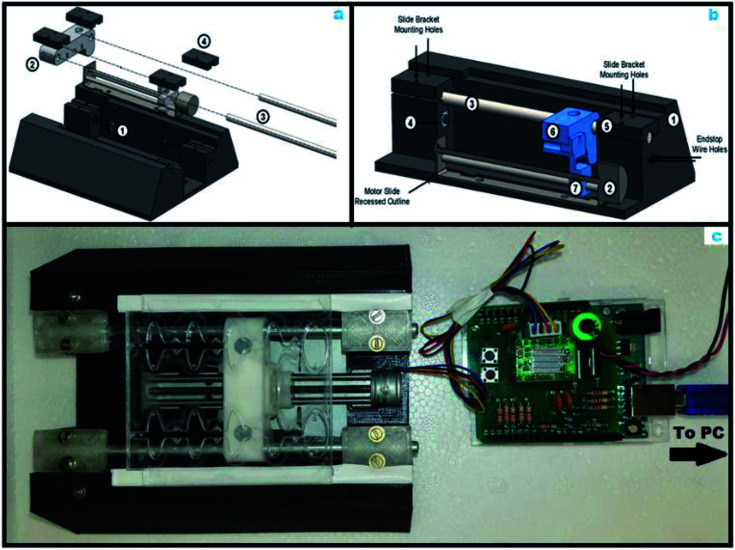
(a–c) The total Mechanical Actuation Platform (MAP) and PCB driver; (a) overall setup of working MAP for the automation of the microfluidic-ELISA; (1) end stop used to signal final resting position to actuator. (2) Magnet housing adaptor for 5 mm magnets. (3) Guiding rails constructed from 3/16 inch stainless steel rods. (4) The rod clamps were 3-D printed with black PLA and fastened with M0.7 screws; (b) cross section of the actuator hardware. Moving parts are in blue. The stepper motor enclosure consists of parts 1 and 5, and the chip itself rests on the groove in part 1. The linear actuator is composed of parts 2, 3, and 4. Parts 6 and 7 form the moving carriage holding the magnets; (c) the MAP consists of nearly all 3-D printed part from PLA, and is assembled with steel rods and screws. The PCB consist of an Arduino shield that houses a Pololu A4988 stepper motor driver with two manual push buttons for both automatic and manual control of the MAP. The Arduino is powered by an external power supply and connects to a PC to run the stepper commands in gcode.

We developed a magnetic actuator to automatically move the microparticles through the microfluidic chip (Fig. 3a). We designed the actuation system to be able to accommodate any microfluidic chip that can fit into a 70 mm × 80 mm × 10 mm volume. The system was designed around a stepper motor, linear slide, and a pair of 5 mm neodymium magnets. The microfluidic chip was aligned with the magnets by a 3-D printed enclosure and the magnets were held in place by a carriage on the linear slide rails (Fig. 3b and c). The electronics consisted of a microprocessor, a stepper motor driver, and power supply circuits.

The hardware diagram (ESI Fig. 1†) and circuit schematic (ESI Fig. 2†) were created to optimize a printed circuit board (PCB) Arduino shield input for software control integration.

Two end stops were added to prevent damage to the stepper motor, and buttons were used for manual control of the machine. To keep chip-specific hardware to a minimum, the actuator was automated using a gcode set of scripting commands. These human-readable commands were sent from a computer to the microprocessor through a serial interface. The microprocessor translated the human-readable commands into stepper motor driver commands and controlled the linear slide through the driver. We determined that the beads were actuated by the stepper motor at 25 mm per second and allowed for change in direction for sufficient bead mixing in each well as the pellet was actuated in the forward and backward directions within the reaction well. Each step in the assay also underwent several optimizations regarding incubation times, bead aggregation and pellet speed control. Despite our wide range of chip dimensions and actuation tests, we found that only four commands were required for any actuation sequence. The finite state machine for our controller, including the actuation commands, can be found in ESI Fig. 3.† We developed a Python library to manage the two-way communication between the computer and the device. Our software parses the sequence of commands from a file and sends them to the actuator, while ensuring that all commands are executed. Using this method, a change in microfluidic chip geometry can be accommodated by using a different command file.

### M-ELISA smartphone quantitation method

The bead-based ELISA platform was finally leveraged in a microfluidic platform (M-ELISA) to facilitate the assay automation and was evaluated using an image-based quantitative method that is more suitable for use in resource-constrained settings. This quantitative method can be implemented on a smartphone, allowing data to be collected, processed, and transmitted without conventional laboratory equipment. Images of the microfluidic chip were collected using an Android Galaxy S7 edge (Samsung). After image capture, the portions of the image containing useful output data were identified as regions-of-interest (ROIs) as we previously reported.^24^ This process was completed using both manual and automatic methods. The manual method consisted of a cross-platform user interface that allowed users to select regions of the smartphone image. The automatic method converted the image to the hue-saturation-value (HSV) colorspace, where hue and saturation thresholds were used to obtain an extraction template. Morphological transformations and contour analysis were applied to the template to determine the location and size of the ROIs. To eliminate false-positive ROI detections, the final detections were filtered by size. Both methods tagged the output well of the microfluidic chip as an ROI. After identification, the ROIs were processed using the mean pixel intensity method (MPI). To improve the detection threshold, the ROIs were converted to the HSV colorspace before MPI analysis. The final output of the algorithm was the MPI of the saturation channel of each ROI. The saturation MPI was chosen based on its high correlation with spectrophotometer output, low standard error, and constant dynamic range under various imaging conditions using a 96-well plate with 100 microliters of horse radish peroxidase (HRP) conjugated anti-p24 (Abcam), serially diluted from 1 : 2000, 1 : 4000, 1 : 8000, 1 : 16 000 1 : 32 000 and 1 : 64 000. The plated dilutions were reacted with 50 microliters TMB and stopped after 5 seconds with 50 microliters of 2 N H_2_SO_4_. The plate was then photographed in optimal and suboptimal lighting conditions for precision.

### M-ELISA with mechanical actuation platform (MAP)

Superparamagnetic Sera-Mag SpeedBeads neutravidin-coated magnetic particles (1 micron in diameter, GE Healthcare) were coated with anti-p24 antibody (Abcam). The bead concentration for actuation was initially tested using serially diluted bead concentrations ranging from 125 μg mL^−1^ to 1 mg mL^−1^. The beads were caught in the path of the magnet in the capture well within fifteen seconds of running the platform. To validate the HIV-1 p24 ELISA system, each reagent was tested using a direct and an indirect ELISA with the recombinant HIV-1 p24 target (see ESI Fig. 5 and 6†). Western blots were also performed to validate the binding of the primary and secondary anti-p24 antibodies to the recombinant HIV-1 p24 antigen. Further validation of the functionalized superparamagnetic beads at an optimized concentration at 500 micrograms per mL was performed in a magnetic bead-based ELISA for the HIV-1 p24 recombinant antigen.

The magnetic bead-based ELISA was setup in the prefabricated microfluidic chip, and after the loading process (ESI Fig. 4†), the chip was placed onto the automation platform. The assay is split into 3 distinct phases, and the reaction proceeds by control of an external magnetic field underneath using a motor-controlled slide (see ESI Fig. 4†). Initially, functionalized 500 microgram per mL magnetic particles coated with anti-p24 capture antibody are collected and suspended in 25 microliters of phosphate-buffered saline (PBS), pH 7.4. Then 25 microliters of the sample is mixed with the functionalized beads (500 micrograms per mL) and the mixture is loaded into the first reaction well. Between the diamond reaction phase wells, ovals containing oil prevent any cross-contamination of the reaction through the principle of liquid–liquid phase separation. The second diamond shaped reaction well contains 50 microliters of an anti-p24 secondary antibody for the target (Abcam), with a conjugated horse radish peroxidase (Abcam) to catalyze the oxidation of 3′,3′,5′,5′-tetramethylbenzidine (TMB), a color changing substrate. In the final reaction well, where 50 microliters of the colorimetric substrate is located, there will be a distinct and direct amount of color change from clear to blue in each assay, per the amount of target antigen present in the sample. The beads are then moved out of the well ceasing the color change reaction into the larger oil retention well, and the chip was photographed using a galaxy 7 edge (Samsung), and the analyte is read using a Nanodrop One (Thermo Fisher Scientific) to give absorbance (650 nm) in optical density. Images were then processed using a manual ROI extraction GUI to calculate the mean pixel intensity (MPI) for saturation. The MPI values were measured from the center of the colorimetric chamber channel to obtain a triplicate mean of saturation MPI.

## Results and discussion

### Magnetic and microfluidics model

We modeled the magnetic field of the permanent neodymium magnet using the remnant flux density measurement from the manufacturer (Br = 14 800 gauss) using COMSOL and solved Maxwell's equations for the magnetic field in our microfluidic chip. We computed the force on the beads using the simulation results and an experimentally verified equation for the force on a magnetic particle in an applied magnetic field (ESI eqn (1)†).

**Fig. 4:**
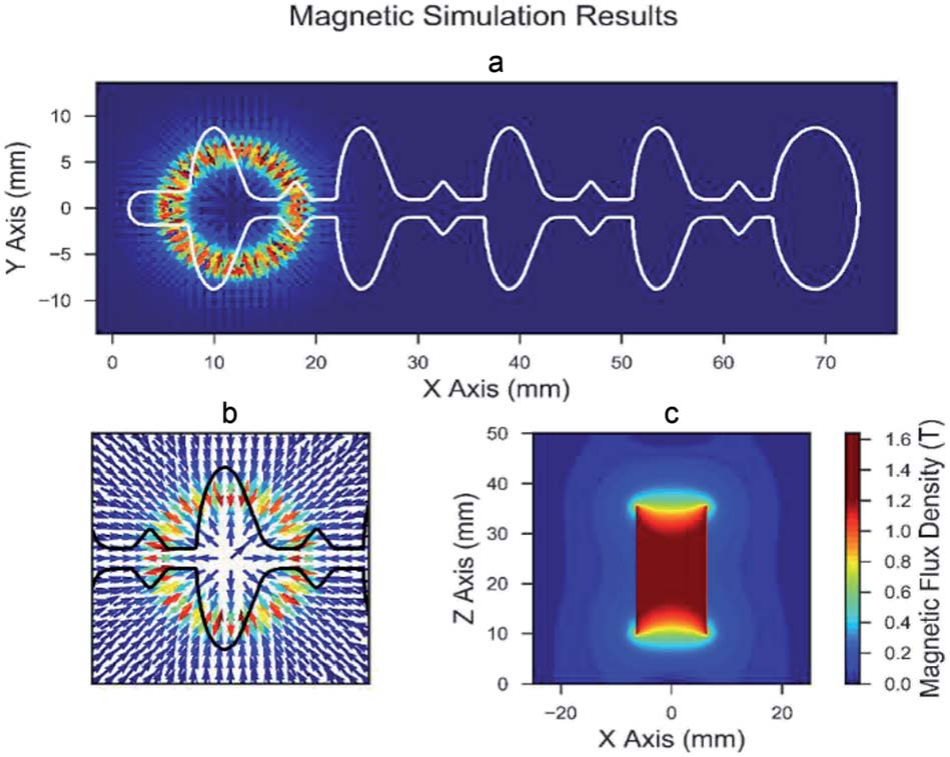
(a–c) The magnetic model results; (a) the calculated results for the magnetic force on a single bead overlaid by the microfluidic chip (Chip D) geometry; (b) the detailed map of magnetic forces, suggesting stronger forces acting on the superparamagnetic beads are found on the edges of the circular external magnet where there is greater change magnetic flux density; (c) a vertical slice map of the magnetic field generated by the 5 mm neodymium magnet demonstrating the greater change in magnetic flux density at the edges of the external magnet.

**Fig. 5:**
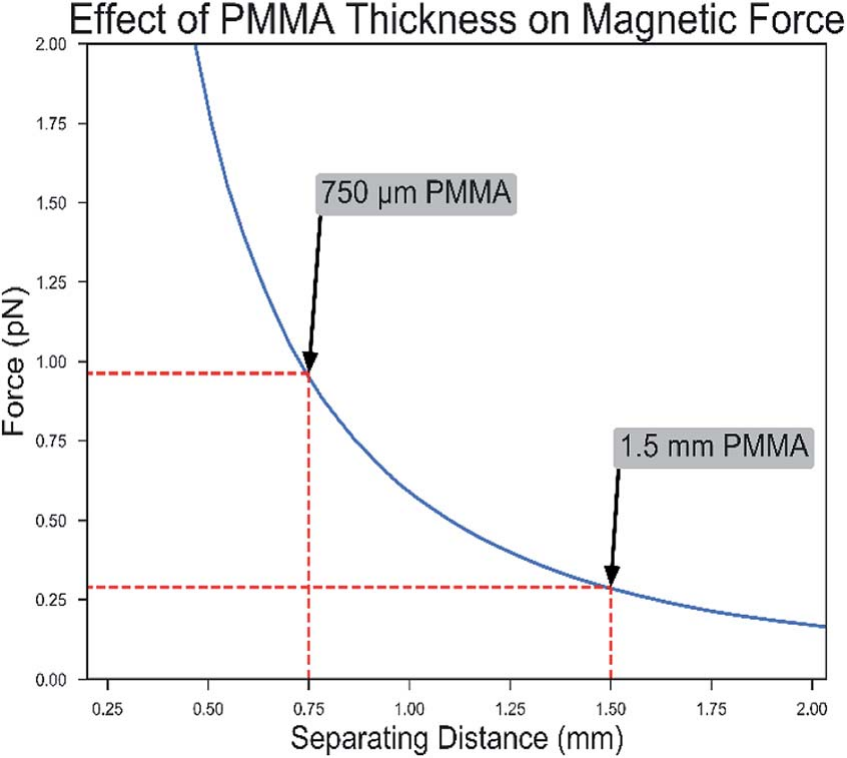
The peak magnetic force *vs.* distance from the magnet; this graph shows the analytical result for the difference in force on one bead when placed at a varying distance to our magnet. This corresponds to conditions of both 0.75 and 1.50 mm PMMA bottom sheets. The force applied with a 0.75 mm sheet is approximately four times larger than the force applied using a 1.5 mm sheet. This allows for better control of the beads, which was evident in the latter optimized microfluidic chips during actuation testing.

ESI eqn (1)† shows that the force is proportional to the gradient of the magnetic field strength, and the magnetic field simulations show that the greatest change occurs at the fringe of the magnetic field (Fig. 4a–c). Our calculated results show that the force is strongest near the edges of the magnet. The results also show that the maximum force on the particles decreases rapidly with separating distance to the chip (Fig. 5). Therefore, we choose 0.75 mm and 1.5 mm thick PMMA for bottom chip layer and well depth respectively, contributing to total chip volume of 529 μL. These selected dimensions of the microfluidic chip allowed the beads to travel through the chip with minimal bead loss, enabling reliable and optimized quantitation. The fluid interfaces and pressure differences within the chip were estimated by solving the Navier–Stokes equations for laminar flow (ESI eqn (2)†). We initialized the simulation with the well volumes used to load the microfluidic chip (ESI Table 1†) and calculated the steady-state position of the fluid phase interfaces. We accounted for the surface tension between PBS and mineral oil (*γ* = 50 mN m^−1^ (ref. 36)) and the interaction of the PBS with the PMMA chip walls (contact angle = 68° (ref. 37)). The simulations show that the designed capillaries (rectangular channels between wells) prevent overflow from one well into another, resulting in a smaller interface with less curvature. We observed that during the bead aggregation stage, the beads aggregated along the edges of the magnet rather than into a clump in the center.

**Fig. 6:**
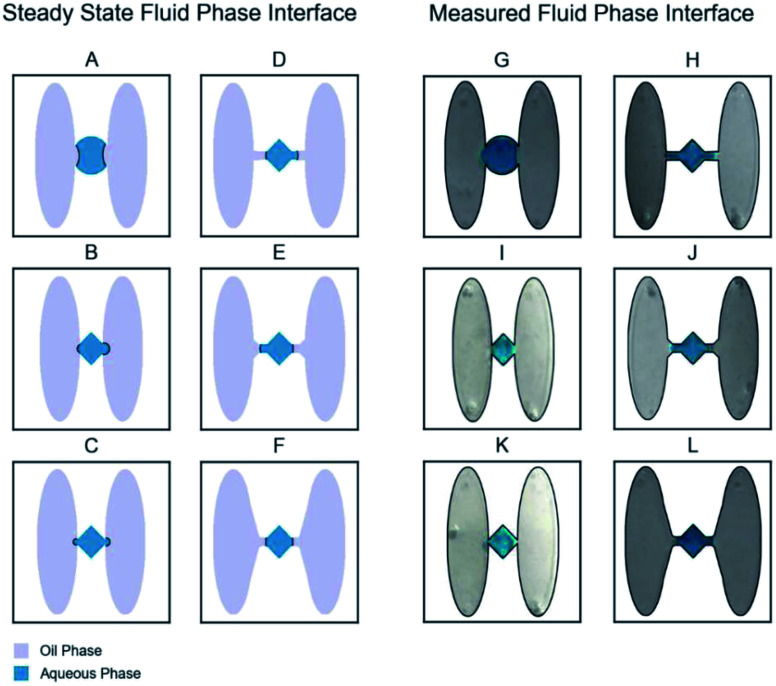
Simulated fluid phase interfaces for six microfluidic designs and the measured effect of chip geometry on oil–water interface. The junctions were truncated to demonstrate the pertinent studied interfacial energies between the oil and aqueous wells; the peak pressure difference across the interface was 6.9 pascal (Pa), 25.6 Pa, 41.0 Pa, 13.0 Pa, 5.6 Pa, and 5.0 Pa for (A), (B), (C), (D), (E), and (F) respectively. Their real-world counterparts showed the expected effect with the letters (G), (I), (K), (H), (J), and (L), when the simulated chip interphases were tested experimentally in the lab using blue dye and oil.

The pressure difference between the two phases was lowest for the design with capillaries and diamond well shapes (fillets), while the pressure difference was greatest for designs without the channel and with fillet modification (Fig. 6). The fillet shape well was chosen because it is easiest to load using a pipette. The simulations show that highest-pressure of 25.0 Pa and 41.0 Pa is generated between oil–aqueous wells when channel capillaries are not included between the wells (Fig. 6B and C) the fillets. However, this high pressure is mitigated when capillaries are added, the pressure difference drops to 13 Pa and 5.6 Pa in Fig. 6D and E, and to 5.0 Pa in Fig. 6F with additional contouring to the adjacent oil wells. The pressure drop is accompanied by a reduction in the curvature of the oil–water interface and an increased tendency for fluids to remain within their wells as also verified with experiments performed in the lab using these designs. We observe similar behavior of oil–aqueous interfaces experimentally as simulated by COMSOL (Fig. 6G–L).

### Microfluidic chip design

The design phase of this project showed progression through the challenges caused by bead retention-related issues (biofouling), where magnetic particles would fail to cross the interface between the aqueous and oil phases. Bead retention causes unpredictable bead loss at every interface and poses problems because a substantial number of beads may be lost, particularly for low magnetic bead concentrations. Thus, it is an issue for the control of the functionalized magnetic particles performing in multiphase microfluidic chips and is problematic because random bead loss prevents reliable actuation and quantitation. This challenge has been experienced in other microfluidic chips^34^ (including our initial well designs in Fig. 6G and H), and our optimized chip (Fig. 6L) addresses the bead retention issue by both containing and physically reducing the interfacial energy barrier within introduced channel capillaries, enabling quantitative assays with low bead concentrations. The design was inspired by the observation that oil–water interfaces with high curvature were more likely to retain beads. Such interfaces produce a high-pressure difference between wells (ESI eqn (2)†), making bead transport more difficult. The optimized chip geometry imposes limitations on the shape of the interface, resulting in a lower pressure difference.

These capillary-style chips are also easier to load by hand, since they provide a buffer that protects against over and under filling, and external vibrational or shifts from motion. There was a risk of spilling reagents from a fully filled chips due to shifts in chip position and when moving the filled chip from the benchtop to the actuation platform. For instance, if the oil well is slightly overfilled, the interface will move horizontally but will not change shape, and the chip can be moved from benchtop to the platform carefree. In the optimized chip design with capillaries, the fluids could shift within the confines of the capillary inlets, preventing reagent spillage or loss of reagent confinement. The implementation of capillary inlets and diamond-shaped aqueous wells (Chip D in Fig. 2) was a great success in the versatility of the platform. The capillary inlets were placed to join the aqueous and oil wells together and acted to further facilitate bead confinement. The aqueous wells benefited the chip allowing for the easy filling of the well by quickly channeling the aqueous reagents into position, and smaller confinement increased the ability to contain the aqueous reagents within the wells. Despite the magnet being of adequate strength, non-specific binding of beads with well surfaces posed a challenge that required multiple chip redesigns and changes to the antigen capture well geometry. If the beads were unable to be actuated in the chip after ten seconds, they were highly subject to non-specific binding to the PMMA chip and could not be formed into the required paramagnetized pellet for efficient actuation. The first antigen capture well design was enhanced from a circle to a rectangular well (Fig. 2b). This rectangular well shape increased the amount of contact the beads had with the applied magnetic field by forcing a physical boundary for the beads that fully utilized the linear path of the strongest magnetic force. These design modifications optimized the bead paramagnetization significantly and prevented the initial limitation of non-specific absorption of the beads to the PMMA chip surface due to the increased response to the magnets and quicker bead aggregation, by focusing the bead solution directly in the path of the magnet, enhancing both the interaction and paramagnetization influence. Additionally, the oil–water interfacial boundary also required optimization as well. The amount of space that forms this “interfacial window” determines the nature of the interfacial energy that is developed. When a smaller channel height is used, the amount of interfacial energy is distributed over less space, which results in a smaller displacement of interfacial energy and a greater boundary force. This was the case when the height of chip wells was decreased to a 0.750 mm, the interfacial energy barrier proved too great for passage of the paramagnetized pellet. However, when the well height was increased to 3.125 mm, the interfacial boundary between the oil and water wells was weakened, leading to loss of chip containment. This was due to increased area of the interfacial boundary contact from the increased channel height. When the height was increased, a greater amount of the interfacial energy is distributed over greater space, leading to the diminished overall interfacial boundary force and reagent confinement issues. This balance was remedied when a 1.5 mm channel height was used, which leveraged just enough interfacial energy between the oil and water interface to confine the reagents, and to equally facilitate the bead pellet to pass through to the next reagent well.

### Platform automation and bead control

Most microfluidic actuators are either manually controlled or application-specific. This automatic system is a step toward a truly general platform that can be used for any chip. By encoding the actuation procedure as a series of executable commands, three key advantages can be obtained: flexibility, standardization, and parallelization. The system is flexible because new microfluidic chip designs can be used with existing hardware, provided the overall dimensions fit the specifications of the machine. Since the actuation procedure is completely reproducible from the command file, the assay is also self-documenting. A user only needs the standard command file and well loading information to run the assay. Changes to the assay can be tracked by changes to the command file, allowing different actuation procedures to be compared. Users can also manage many assays simultaneously since user interaction is only required at the beginning and end of a test. There was a fine balance between generating optimal adequate magnetic field strength and the concentration of functionalized beads used. The beads followed a linear path along the chip where greatest force was applied on the edges of the circular magnet. It was found that bead capture is dependent on the applied magnetic field strength, which decreases rapidly with distance from the magnet (Fig. 5). Therefore, both 1.50 mm and then 0.75 mm PMMA were used for the base of the chip. The optimal bead concentration was found off chip in a magnetic-bead based ELISA, and a series of standards were tested to develop a standard curve. Using the optimized concentration for beads, reagents, and optimized chip design, the actuation platform was used to automate the M-ELISA. The exact bead position depended on the number of steps given to the stepper motor driver from the user-generated gcode. Depending on the chip design, the gcode varied, however each design was easy to configure due to the repeated geometries and position of the chip wells. We also provided a mixing scheme by optimizing the control of magnetic actuation (through use of a g-code controlling a stepper motor), and by optimizing the liquid–liquid interphase between the reagent and oil wells by incurring a lower surface energy due to the optimized shape and size of the fluid interface geometry and bead drag reduction using a linear extension of the aqueous well. This allows the actuation platform to be easily accommodated to any assay-type that can operate on a similar linear track within the same spatial confines. Furthermore, the adoption of the M-ELISA demonstrates the versatility of the platform to adopt complex assays. On occasion, if the pellet was overexposed to the oil phase, it led to failure of bead actuation. The oil phase serves only to provide reagent well separation; however, when passing beads through at a slow or unreliable speed has detrimental effects to the assay. We hypothesize on the molecular level, when oil forms micelles along the boundary with aqueous well, there can be issues associated with the beads passing through, and the micelles favorably form around the hydrophilic bead aggregate surface becoming encapsulated in oil. Specifically, with this detrimental effect, the oil-encapsulated beads are then unable to react with the further downstream reagent, or the oil-encapsulated beads would be attracted to the hydrophobic PMMA surface and prone to biofouling as the beads were pulled along the assay. This would become more apparent in the latter parts of the assay, after the beads had passed through multiple oil phases at suboptimal speed. During each incubation and wash step the beads were continuously moved in the forward and backward direction in the wells to remove oil encapsulation and to enhance the assay reactions. We found that the optimal speed of bead aggregate was 25 mm s^−1^, at this speed the beads would be able to penetrate through the oil phase wells in 0.5 seconds (12.5 mm channel length) and have reduced exposure to the oil.

Refinement of the gcode to match the desired position of the beads is also crucial to prevent exposure to potential non-specific binding. One other aspect that can be explored in the future would be the use of lower viscosity oils or oil blends, as this could reduce the interfacial energy barrier, further facilitate bead passage, and potentially lower the exposure of beads to oil.

### Automated microfluidic-ELISA

We performed several validation steps for our reagents sets including direct ELISA (ESI Fig. 5†), indirect ELISA (ESI Fig. 6†), western blot analysis (ESI Fig. 7†) of the antibody and target binding. Results reported in ESI Fig. 5–7† clearly shows that the selected anti-p24 antibodies showed reactivity and binding to p24 antigen. Western blot results (ESI Fig. 7†) show that selected antibodies do not show any cross-reactivity with negative control (dengue virus type 3).

**Fig. 7:**
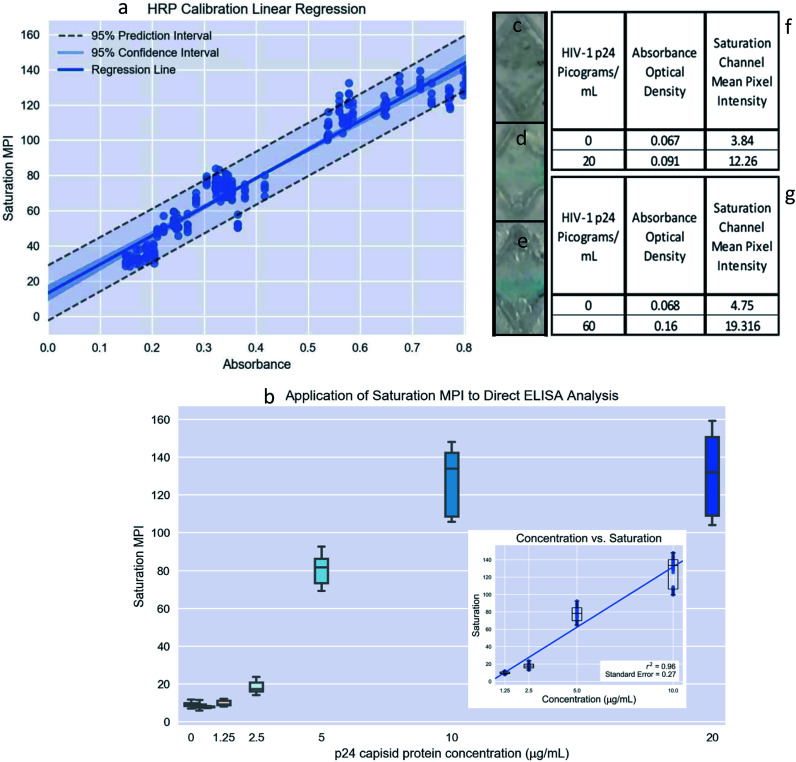
(a) The 95% C.I. graph of absorbance (OD) and corresponding smart phone quantitation method using saturation mean pixel intensity (MPI) for an HRP calibration curve; (b) the use of the smart phone quantitation method on an HIV-1 p24 ELISA showing a limit of detection at 1.25 μg mL^−1^; inset: linear regression of concentration and saturation inside the linear response range for saturation with *r*^2^ value = 0.96 (between 1 μg mL^−1^ and 10 μg mL^−1^). Additionally, a 1-way ANOVA was performed for all pairs of concentrations analyzed. We found that the difference in saturation response is statistically significant for all pairs of concentrations between 0 μg mL^−1^ and 10 μg mL^−1^. (c–e) (top) 0, (middle), 20 and (bottom) 60 picograms per mL results from the recombinant HIV-1 p24 automated M-ELISA using the OD and cell phone quantitation method; (f and g) the negative controls tested had an average saturation MPI 3.84 and 4.75, which corresponded to average absorbance optical density (OD) values of 0.067 and 0.068, respectively. The average saturation MPI values at the target concentrations were determined to be 12.26 for 20 pg mL^−1^ and 19.316 for 60 pg mL^−1^ of recombinant HIV-1 p24, corresponding to average OD values of 0.091 and 0.160, respectively.

To show the application of the developed platform, microfluidic ELISA (M-ELISA) was performed to determine concentration of HIV-1 p24, with the run time for each assay being twenty minutes. The M-ELISA was tested at a concentration of 20 pg mL^−1^ and 60 pg mL^−1^ (low and mid-range p24 targets) showing successful demonstration of the microfluidic chip automated platform (Fig. 7a–g). The smart phone quantitation algorithm was calibrated (Fig. 7a) with the HRP calibration curve using several ROIs. The extracted ROIs yielded saturation MPI values from smart phone images for the negative controls and different target concentrations to give an average MPI value for each sample individually. Precision and robustness of the smart phone quantitation method was tested using the anti HIV-1 p24 direct ELISA (Fig. 7b). The negative controls tested had an average saturation MPI 3.84 and 4.75 (Fig. 7c), which corresponded to average absorbance optical density (OD) values of 0.067 and 0.068, respectively. The average saturation MPI value at the target concentrations were determined to be 12.26 for 20 pg mL^−1^ (Fig. 7d) and 19.316 for 60 pg mL^−1^ (Fig. 7e), corresponding to average OD values of 0.091 and 0.160 (Fig. 7f and g), respectively.

The major focus of the project was to develop a potential POC platform that can be utilized for an immunoassay. However, in the future we plan to also investigate a linear range over a wider number of target concentrations with additional specificity and whole blood studies. The microfluidic platform can be adopted for any microfluidic ELISA-based detection and is automated, which demonstrates its versatility. In comparison with 96/384 well plate, the throughput of the developed platform is lower however its intended use is for POC settings in developing countries where we may need to run only few samples per day, this platform can do the job at much lower cost. Multiple assays can be made to run simultaneously if needed, with design modification. Using colorimetric smart phone-based detection, the end-user can be easily enabled to become a self-tester and diagnose potential illness in POC situations.^24,25^ This platform has the potential to be adapted to a multitude of potential pathogens or physiological ailments detected previously through ordinary ELISA testing. Thus, the developed automated microfluidic-ELISA has potential to be considered for POC disease testing and future adaptability for the next generation biosecurity measures, preventative POC diagnostics in disease epidemics, and for routine clinical monitoring at the patient's bedside.

## Conclusions

The developed M-ELISA chip is cost-effective as it uses low-cost acrylic plastics for fabrication and requires small reagent microfluidic volumes. With the quick assay turnaround and quantitative result, the developed platform is beneficial for potential POC applications. The automated microfluidic ELISA platform was validated using the HIV-1 anti-p24 M-ELISA model. Using the M-ELISA chips integrated with actuation platform, we show that we can detect clinically relevant concentrations of p24 antigen within twenty minutes of assay runtime. The whole process is automated which is a significant improvement and platform does not require highly skilled labor to operate. Thus, a highly versatile automated platform for a magnetic bead-based microfluidic ELISA was developed for the detection of p24 antigen, which can be further developed for rapid detection of other viral and bacterial pathogens, or for maintenance of chronic illnesses through routine detection at home or the POC.

## Conflicts of interest

There are no conflicts to declare.

## Supplementary Material

RA-009-C8RA07607C-s001
