# Supplementary material for: Development of a flow-free magnetic actuation platform for an automated microfluidic ELISA
Source: RSC Adv. 2019 Mar 12;9(15):8159–68. doi: 10.1039/c8ra07607c (PMC6880949; doi:10.1039/c8ra07607c)
Supplement: RA-009-C8RA07607C-s001 [file RA-009-C8RA07607C-s001.pdf]

Electronic Supplementary Material (ESI) for RSC Advances.
This journal is © The Royal Society of Chemistry 2019

Supplemental Information

Development of a Flow-Free Magnetic Actuation Platform for an Automated Microfluidic ELISA

Chad Coarsey, Benjamin Coleman, Md Alamgir Kabir, Mazhar Sher, Waseem Asghar

Supplementary Table 1: Comparison of Optimized Microfluidic Chip Dimensions; The chip layer thickness measurements of the various optimized chip prototypes and each chip well Volume measurements, with first, Chip A and Chip D being the final prototype.

Chip Thickness and volumetric dimensions

Comparison of Optimized Microfluidic Chip Dimensions				
Chip Layer Thickness Measurements (micrometers)				
Layer	Chip A	Chip B	Chip C	Chip D
Top (Loading)	1500	1500	750 or 1500	750 or 1500
Middle (Well)	3125	3125	1500	1500
Bottom (Base)	1500	1500	750	750
Chip Well Volume Measurements (microliters)				
Well Type	Chip A	Chip B	Chip C	Chip D
Capture	191.94	82.06	39.39	29.67
Aqueous	112.88	37.63	26.18	31.16
Separation (oil)	503.66	211.375	103.89	116.52
Retention (oil)	862.19	361.84	173.69	173.69

Supplementary Table 2: List of costs of materials and tasks required for chip fabrication and assay testing

Assay and Platform costs

MICROFLUIDIC CHIP / ASSAY ELEMENTS		
**Item/Task for Assay and Platform**	**Cost ($)**	**Time Required (min)**
Poly(methyl methacrylate) (PMMA)	$0.1	N/A
Double Sided Adhesive ((DSA)	$0.1	N/A
laser cutting	N/A	1 minute per chip
Chip reagent/oil loading	$0.6	3 minutes
Chip Assay Runtime	N/A	20 minutes
TOTAL COST	$0.8 per test	24 minutes total assay
PLATFORM ELEMENTS		
**Item/Task for Assay and Platform**	**Cost ($)**	**Time Required (min)**
Arduino Uno R3 Microcontroller	$7.00	N/A
Zip ties	$0.15	N/A
Screws	$0.50	N/A
Aluminum rails	$1.50	N/A
1/2 x 1/8 Inch Neodymium Disc Magnets N48	$3.00 (6 per chip)	
3-D printing	N/A	300 per platform*
TOTAL COST	$12.15 per platform	300 per platform
*One-time cost of 6 hours to print the platform (not included in total)		

Circuit Schematic and System Diagram

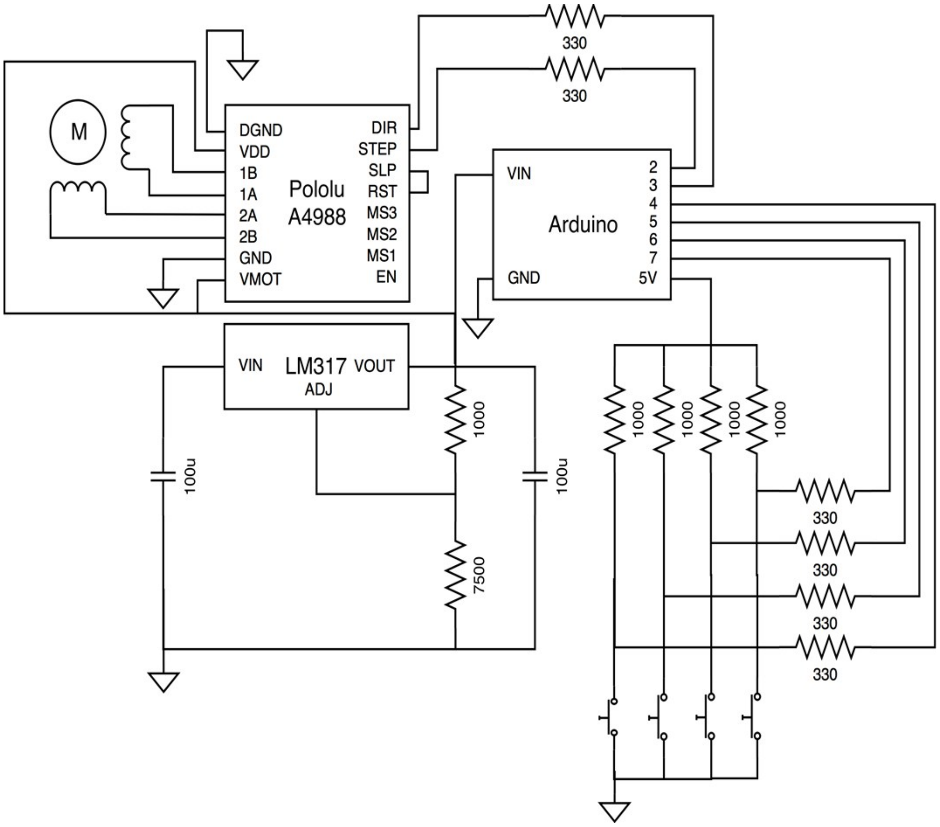

S1: System diagram of the magnetic actuator; To facilitate rapid chip development, the system was designed as a collection of modules with well-defined interfaces.

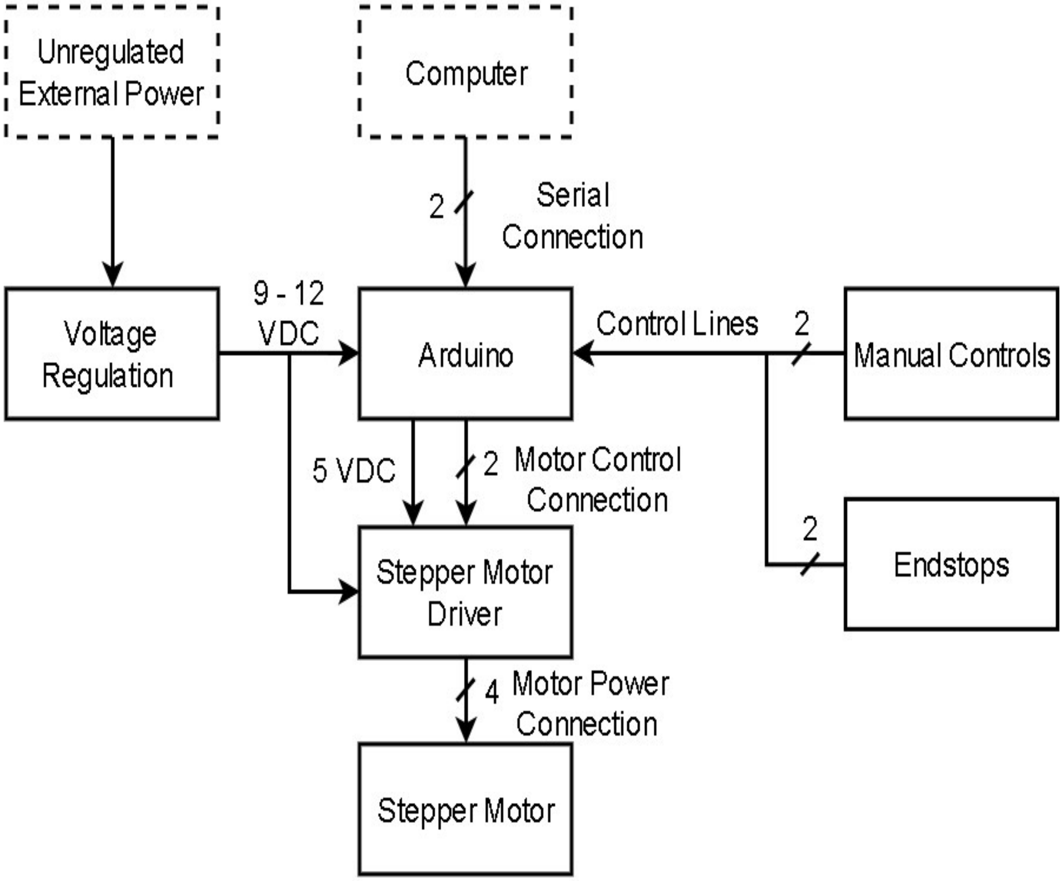

S2: Circuit schematic for the actuator hardware

Platform Control Diagram

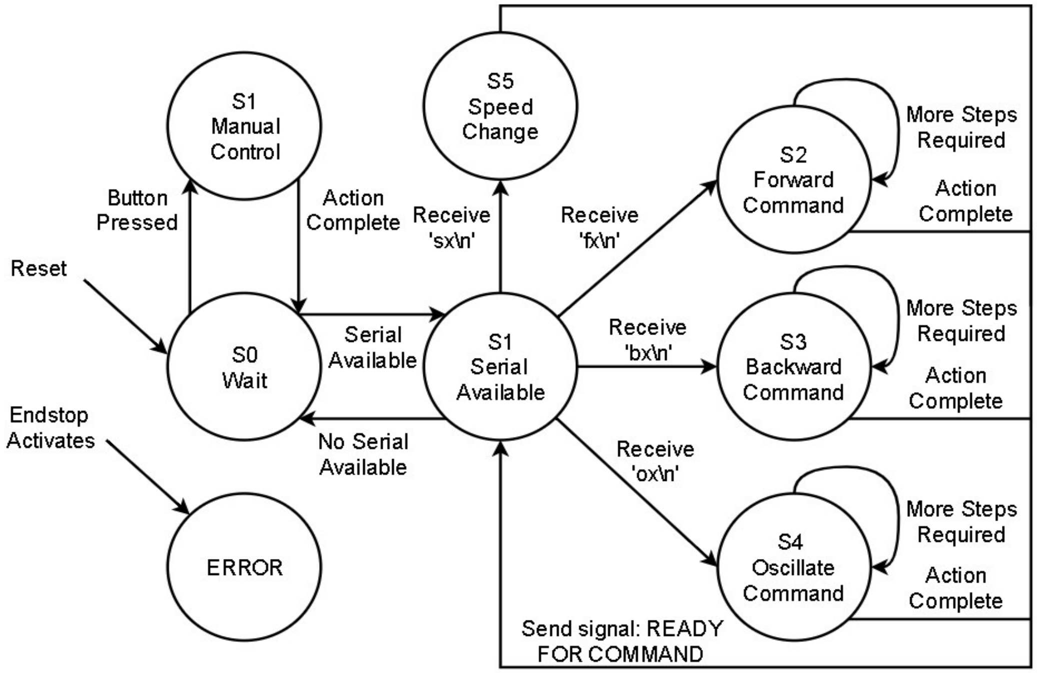

S3: Diagram for actuator control over serial. The Arduino begins in S0 and waits for a serial connection or manual input. If a connection is present, the algorithm waits for a newline-terminated command, dispatches the command to the hardware, verifies that the command has been executed, and signals to the driver that it is ready for the next command.

Chip Loading Diagram

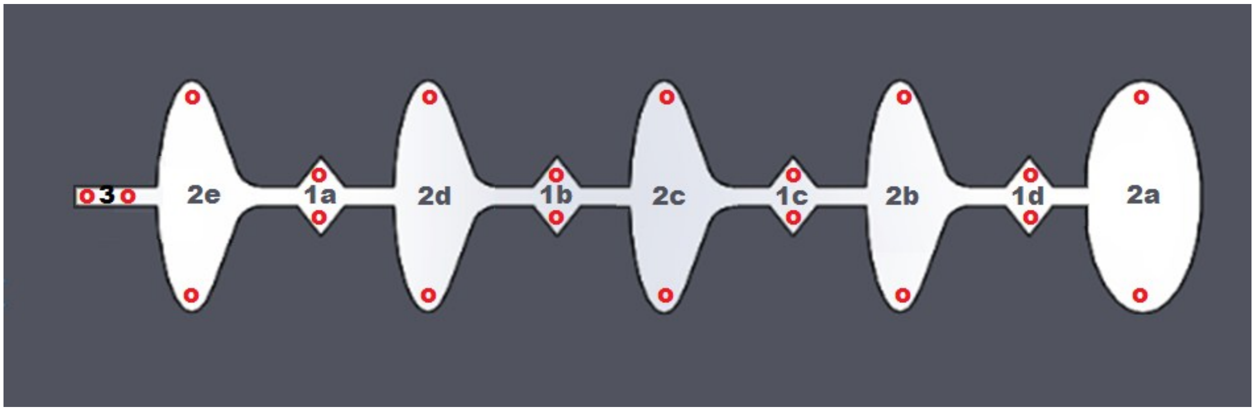

S4: The diagram above shows the alphanumeric order of chip loading; 1a-Wash buffer (PBS), 1b-secondary antibody/HRP conjugate, 1c-PBS, 1d-TMB colorogenic solution, 2a-mineral oil, 2b-mineral oil, 2c-mineral oil, 2d-mineral oil, 2e-mineral oil, 3-primary antibody-coated bead solution and target; Red circles indicate loading and pressure release holes.

Magnetic Force Simulations

Supplemental Equation 1 is the conventional expression for the force experienced by a magnetic particle in an applied magnetic field.

$$\mathbf{F} = \frac{V \Delta \mathcal{X}_v}{\mu_0} (\mathbf{B} \cdot \nabla) \mathbf{B} \qquad (1)$$

F is the force exerted by the field on the particle (newton), V is the particle volume (m^3), X_v is the dimensionless magnetic volume susceptibility, μ_0 = 4π * 10^-7, and B is the magnetic flux density (tesla).

X_v was obtained from the magnetization curve provided by the nanoparticle manufacturer using a standard method. V was directly specified by the manufacturer.

Supplemental equation 1 was implemented in COMSOL using the partial differential equation (PDE) module. A stationary simulation was performed on a model of the permanent neodymium magnet (K & J Magnetics) using COMSOL's magnetic-fields-no-current interface. The results were used with the PDE module to obtain the partial spatial derivatives of the convective operation in Equation 1. Results were exported and post-processed in Python to obtain the final force results for the beads.

Fluid Simulations

The pressure difference at the interface of two immiscible fluids can be found using Supplemental equation 2 when there is no fluid flow. Since our microfluidic chips do not use any flow and our simulations run until steady-state conditions are achieved, Equation 2 is an accurate description of the pressure differences on either side of the oil-water interface.

$$\hat{p} - p = \sigma \nabla \cdot n \qquad (2)$$

p and $\hat{p}$ are the absolute pressure values on either side of the interface, σ is the surface tension coefficient, and $\nabla \cdot n$ is the curvature, where n is the unit normal vector to the interface and ∇ is used as shorthand for the divergence operation.

We computed the pressure fields and steady-state interface locations for our microfluidic chip using the level set method in COMSOL. We used the laminar flow interface and set the initial conditions to the well loading volumes used for the assay.

HIV-1 p24 Target Validation

Direct anti-p24 ELISA: p24 antigen Target Validation

Using a recombinant p24 antigen protein (Abcam), a series of seven (7) two-fold dilutions were used respectively in Rows A-H: 20 micrograms/mL, 10 micrograms/mL, 5 micrograms/mL, 2.5 micrograms/mL, 1.25 micrograms/mL, 0.625 micrograms/mL, 0.3125 micrograms/mL. Each antigen target was diluted in a carbonate buffer (pH 10) (Fisher Scientific) then loaded into the 96-well plate. The plate was covered with parafilm wax and left to incubate overnight at 4 degrees C. The plate was washed with Phosphate-Buffered Saline (PBS) (pH 7.0) 3 times and tapped on Kim Wipes (Fisher Science) between each rinse to empty the wells completely. The plate was then washed with PBS (pH 7.0) three (3) times, and tapped on between each rinse to empty the wells completely. The plate 30 had 100 microliters of blocking solution (Thermofisher) added to each well, and the plate was covered with parafilm wax, and left to incubate overnight at 4 degrees C. The plate was washed with PBS (pH 7.0) four (4) times, and tapped on Kim Wipes (Fisher Science) between each rinse to empty the wells completely. The anti-p24 conjugate (Abcam) was diluted to 0.2 micrograms/mL (1:5000), and 100 microliters was added to each well, and incubated at room temperature for 1 hour. The plate was washed with PBS (pH 7.0) three (3) times, and tapped on Kim Wipes (Fisher Science) between each rinse to empty the wells completely. The plate was developed with the TMB substrate (Thermofisher) with 100 microliters to each well, and stopped 100 microliters with 2M H2SO4 after five minutes to each well. The plate was read by spectrophotometer at 450 nm (SpectraMax)

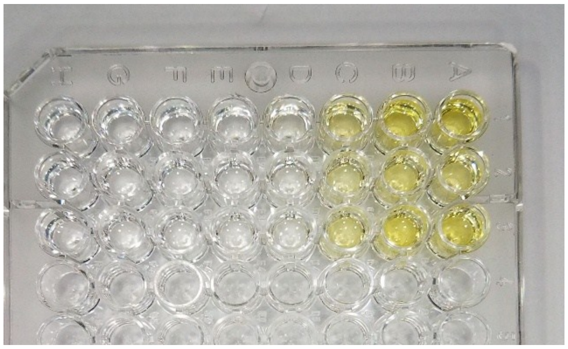

S5: The Anti-p24 Direct ELISA;

Indirect anti-p24 ELISA: anti-p24 Capture Antibody Validation

Using the same recombinant p24 antigen protein (Abcam), a series of seven (7) two-fold dilutions were used respectively in Rows A-H: 20 micrograms/mL, 10 micrograms/mL, 5 micrograms/mL, 2.5 micrograms/mL, 1.25 micrograms/mL, 0.625 micrograms/mL, 0.3125 micrograms/mL, Blank. Each antigen target was diluted in a carbonate buffer (pH 10) (Fisher Scientific) then loaded into the 96-well plate. The plate was covered with parafilm wax and left to incubate overnight at 4 degrees C. The plate was washed with Phosphate-Buffered Saline (PBS) (pH 7.0) 3 times and tapped on Kim Wipes (Fisher Science) between each rinse to empty

the wells completely. The plate was then washed with PBS (pH 7.0) three (3) times, and tapped on between each rinse to empty the wells completely. The plate had 100 microliters of blocking solution (Thermofisher) added to each well, and the plate was covered with parafilm wax, and left to incubate overnight at 4degreesC. The plate was washed with PBS (pH 7.0) four (4) times, and tapped on Kim Wipes (Fisher Science) between each rinse to empty the wells completely. The anti-p24 antibody (Abcam) was diluted to 0.2 micrograms/mL (1:5000), and 100 microliters was added to each well, and incubated at room temperature for 1 hour. The plate was washed with PBS (pH 7.0) three (3) times, and tapped on Kim Wipes (Fisher Science) between each rinse to empty the wells completely. 100 microliters of diluted anti-mouse conjugate (Abcam) (1:1000) to each well, and incubated for 1 hour. The plate was washed 4 times with a permanent magnet using PBS-T and discarded the supernatant. The plate was developed with the TMB substrate (Thermofisher) with 100 microliters to each well, and stopped 100 microliters with 2M H2SO4 after five minutes to each well. The plate was read by spectrophotometer at 450 nm (SpectraMax)

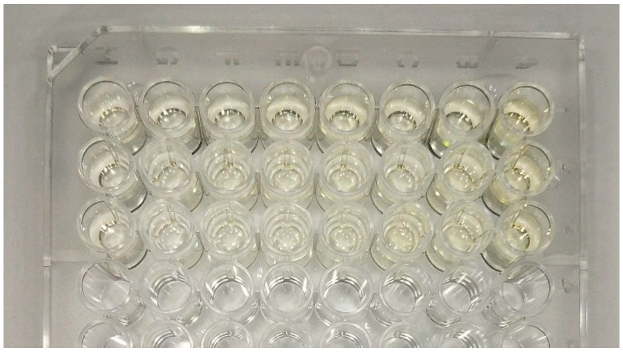

S6: The Anti-p24 Indirect ELISA;

Anti-p24 Western Blot for Antibody Validation

The recombinant p24 antigen protein (Abcam) was used to make serial dilutions of 0.25 micrograms/microliter, 0.125 micrograms/microliter and 0.0625 micrograms/microliter. Each antigen was diluted of PBS (pH 7.4) (Fisher Scientific) then lysed with the lysis buffer. After vortexing for 30 seconds it was placed on the heater for 5 minutes at 95 degrees C. Then it was centrifuged at 13000 rpm for 10 mins and then the samples are ready to load. The glass plates were then set up on the rack of the electrophorator (Amersham Biosciences) following by preparing 13% running gel using ddH2O (10.4ml), 40% acrylamide (Thermofishcer) 8.1 ml, 1.5 M Tris (pH 8.8) 6.25 ml, 10 % SDS 250 microliter, 10% ammonium persulfate 250 microliter and TEMED (Thermofisher) 40 microliter. After pouring the running gel on the glass plate 2 ml of Butanol (Thermofisher) was added to make the top layer of gel straight. Once the TEMED was added, the gel starts polymerizing and after 25 mins the top layer was rinsed with water and make dried using blot paper. The comb was set up and stacking gel was poured on top of the running gel. The stacking gel was prepared using ddH2O (3.13 ml), 40% acrylamide (Thermofisher) 0.625 ml, 1.5 M Tris (pH 6.8) 125 ml, 10% ammonium persulfate 50 microliter and TEMED (Thermofisher) 12-15 microliter.

After polymerization, the comb was removed and the samples (40 ul in each lane) with ladder and control was loaded on to the gel. Running buffer (30.0 g of Tris base, 144.0 g of glycine, and 10.0 g of SDS in 1000 ml of H_2O with adjusted pH 8.3 in total volume of 10L) was poured into a gel box prior to electrophoresis. The power supply (Bio-Rad) was connected to the rack and turned on at 220V voltage & 60 micro Amps current; the runtime was 3.25 hours. After the electrophoresis the stacking gel was discarded, and the separated gel part was used to transfer the proteins on the membrane. A transfer sandwich was prepared using 2 blot papers (Thermofisher), Nitrocellulose Membrane (GE Healthcare), gel and again the blot paper. Then the was relocated on the transfer apparatus and poured with transfer buffer (Methanol 2 Liter, Glycine 113 g, 6g of SDS,24 g of Tris base and 8L of H_2O). 150V voltage and 400 micro amps current was supplied for 3 hours then it was incubated overnight at 4 degrees C.

After the incubation the membrane was blocked for 15-30 mins with 5% skim milk prepared in Tris-Buffered Saline Tween-20 (TBST) (pH 7.5). Then the nitrocellulose membrane was rinsed 2x with TBST followed by 1-hour incubation on a rotator with the 30 ml of anti-p24 antibody (Ab9071 & Ab19961, Abcam), 3% BSA, and 0.05% Sodium Azide (VWR) solution at room temperature. The membrane was washed 3 times with TBST for 5-10 mins on a rotator. Anti p24 with HRP conjugated antibody was diluted (1:5000) with 2.5% skim milk in TBST and the membrane was incubated 1 hour at room temperature. The membrane was washed 3 times with TBST for 5-10 mins on a rotator. After the third wash, the membrane was incubated for 2-5 mins on TBS (without Tween) and the membrane read by the Odyssey classic imaging system (LI-COR) at 700 and chemiluminescence channel with adding the signal enhancer substrate (Thermofisher).

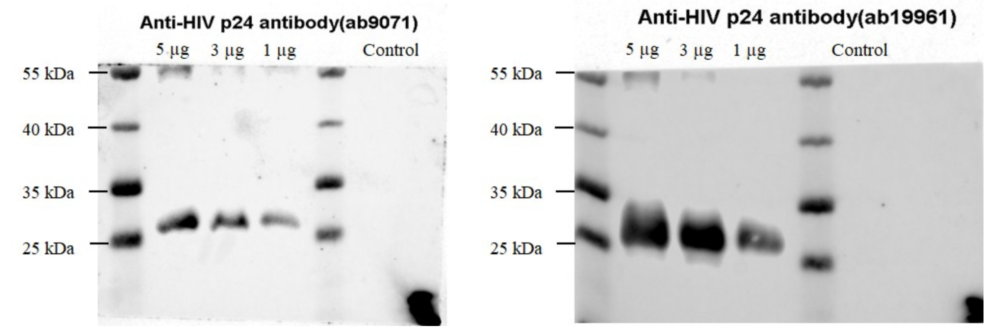

S7: The western blot analysis of both Anti-p24 antibodies (abcam); The primary anti-p24 antibody (ab9071) was run with the recombinant p24 antigen (left gel). The secondary HRP conjugate (unconjugated) was run also with the same recombinant HIV p24 antigen (right gel). The gels showed no banding in the negative control (NR-80 Dengue virus type 3, Philippines/H87/1956), but p24 banding in 5, 3 and 1 ug/mL concentration of each antibody.

Antibody-Bead Functionalization

Superparamagnetic beads coated with an neutravidin molecule (GE Healthcare) allowed for easy surface functionalization of biotinylated capture antibody. Typically, the antibodies used were first needing to be conjugated to biotin. A biotinylation kit (ab20365) was used following

Abcam's protocol to conjugate free biotin with anti-p24 antibody. Following biotinylation of the antibody, avidin coated beads were washed 5 times in 0.05% Bovine Serum Albumin (Thermofisher) solution, and added to the biotinylated antibody at a ratio of roughly 7.6% the full 3500 picomole/mg binding capacity of the avidin on the beads, and left to shake overnight. The beads were left in PBS at 4 degrees after rinsing 5 additional times.
